# Exploring QoS metrics in SDN-based IoUT: A comparative study of 3D topology design techniques

**DOI:** 10.1371/journal.pone.0357016

**Published:** 2026-10-30

**Authors:** Reza Mohammadi, Shohreh Jaafari

**Affiliations:** Department of Computer Engineering, Bu-Ali Sina University, Hamedan, Iran; Guilin University of Technology, CHINA

## Abstract

The Internet of Underwater Things (IoUT) has become a pivotal domain for advancing underwater communication and monitoring systems. However, achieving efficient and reliable communication in 3D underwater environments remains challenging due to constraints such as limited bandwidth, high energy consumption, and severe signal attenuation. This paper investigates Quality of Service (QoS) optimization in Software-Defined Networking (SDN)-based IoUT environments through the implementation and analysis of various 3D topology design algorithms, including Cubic, Hexagonal Prism, Rhombic Dodecahedron, and Truncated Octahedron. These algorithms aim to optimize network connectivity and coverage by determining the spatial arrangement of underwater nodes. The SDN controller computes shortest paths, informs nodes of their routing decisions, and directs packet forwarding. Beyond a direct comparison of existing topologies, this study provides a unified SDN-based evaluation framework to analyze how 3D geometric node placement affects QoS performance, routing efficiency, and deployment cost in underwater IoUT environments. Key QoS metrics, including throughput, network lifetime, energy consumption, and delay, are evaluated and compared across these topologies to identify the most effective design for underwater networks. The results provide valuable insights into the trade-offs between topology structures and their impact on QoS, contributing to the development of more robust and energy-efficient SDN-enabled IoUT systems. The simulation results indicate that the RD method reduces the number of deployed nodes by approximately 63% while concurrently improving network lifetime by 25% and decreasing end-to-end delay by 16%.

## Introduction

The Internet of Underwater Things (IoUT) represents a revolutionary advancement in the field of communication and monitoring systems, offering immense potential for applications such as environmental monitoring, underwater exploration, disaster prevention, and surveillance [1–3]. As an extension of the Internet of Things (IoT) into the underwater domain, IoUT networks aim to connect a wide array of underwater sensors and devices to enable intelligent and autonomous operations in aquatic environments [4]. However, the deployment and operation of IoUT face significant technical challenges due to the unique characteristics of underwater environments, such as high signal attenuation, limited bandwidth, high propagation delays, and energy constraints [5].

Communication in IoUT predominantly relies on acoustic signals, which, despite their suitability for long-range transmission in water, suffer from high latency, limited data rates, and susceptibility to interference [6]. These limitations necessitate innovative approaches to network design and management to ensure efficient and reliable communication. Among these approaches, Software-Defined Networking (SDN) has emerged as a promising paradigm to address the inherent challenges of IoUT [7,8]. By decoupling the control plane from the data plane, SDN enables centralized network control, dynamic resource allocation, and adaptive topology management, making it particularly well-suited for the dynamic and resource-constrained environments of IoUT [9].

In SDN-based IoUT networks, the spatial organization and management of nodes play a critical role in determining overall network performance. Topology control, which involves optimizing the placement and coordination of nodes, becomes especially important in 3D underwater environments where network connectivity, energy efficiency, and communication reliability are directly influenced by node deployment [10]. Efficient topology design can improve key QoS metrics such as data delivery, energy consumption, end-to-end delay, and packet delivery ratio, while also prolonging network lifetime.

Various topology design algorithms have been proposed for 3D underwater environments, each with distinct advantages and trade-offs [11,12]. Among these, geometric configurations such as the Cubic (CB), Hexagonal Prism (HP), Rhombic Dodecahedron (RD), and Truncated Octahedron (TO) have gained attention due to their ability to balance connectivity and energy efficiency while ensuring effective coverage [13]. These configurations offer structured approaches for organizing nodes in 3D space, which can help address some of the unique challenges associated with underwater networks.

This paper investigates the QoS performance of SDN-based IoUT networks employing the mentioned geometric topology design algorithms. By leveraging the SDN paradigm for centralized network control, we aim to evaluate the effectiveness of these topologies in enhancing underwater network performance. A hop-count-based shortest-path routing scheme is utilized to ensure efficient data transmission across nodes, further improving the overall network efficiency.

Although several studies have investigated node deployment strategies in underwater networks, most of them focus on conventional non-SDN architectures and primarily address coverage or connectivity in isolation. A unified analysis of how geometric topology design interacts with SDN-based routing, QoS performance, and deployment cost in 3D IoUT environments is still lacking. In this regard, the contribution of this work is to present an integrated SDN-based evaluation framework that systematically examines how different 3D topology structures influence network behavior under identical routing and simulation conditions.

The primary contributions of this work are as follows:

- **Design and Implementation:** We design and implement distinct 3D topology design algorithms tailored for SDN-based IoUT environments.
- **Integration of Routing Mechanisms:** A global shortest path routing is integrated into the proposed topologies to guarantee data transmission between nodes.
- **Comprehensive QoS Analysis:** We evaluate the performance of the topologies using critical QoS metrics, including packet loss, energy consumption, and delay, and provide a comparative analysis to identify the most efficient configuration.

It is worth mentioning that precise placement of underwater nodes at the exact centers of geometric shapes (e.g., cube, hexagonal prism) is inherently difficult in real-world conditions due to environmental factors such as water turbulence, marine life interference, and vessel movement. However, with the help of underwater positioning and anchoring technologies, it is possible to approximate these placements within acceptable error bounds. The goal of this work is not to enforce perfect node alignment, but rather to advocate for geometry-aware deployment strategies that can improve coverage and QoS compared to random distribution. By using mathematically defined topologies, we aim to show how efficient placement of a sufficient number of nodes can cover a 3D space effectively while avoiding unnecessary or redundant node deployments. The results of this study provide valuable insights into the trade-offs associated with different topology design algorithms and their implications for QoS in SDN-enabled IoUT systems. By identifying the most effective topology for specific operational requirements, this research contributes to the development of robust and energy-efficient underwater communication networks.

Given that 70% of the Earth’s surface is covered by water, underwater networks play a crucial role in environmental monitoring, disaster prevention, and maritime security. The IoUT offers transformative potential for these applications, yet its performance is severely constrained by the unique challenges of underwater communication, including high latency and limited bandwidth. While SDN enables dynamic and efficient management, the impact of geometric node placement on QoS remains underexplored. This study investigates how structured 3D topologies enhance QoS in SDN-based IoUT networks, aiming to improve coverage, energy efficiency, and reliability in critical underwater operations.

The remainder of this paper is organized as follows: Section 1 provides an overview of related work in IoUT and topology design. Section 2 details the proposed topology design algorithms and routing mechanisms. Section 3 discusses the experimental setup, results, and analysis. In Section 4, we give some discussion regarding the node placement in the underwater environment. Finally, Section 5 concludes the paper.

## Related work

Topology design in SDN-based IoUT environments has not been deeply investigated in the existing literature. While SDN has shown promise in addressing the unique challenges of underwater networks, such as centralized control and adaptive resource management, most research efforts have focused on traditional (non-SDN) underwater networks. To provide context, this section reviews key contributions in topology design for non-SDN underwater networks, highlighting the need for further exploration in SDN-enabled IoUT systems.

Topology control has long been recognized as a critical factor in improving network performance and prolonging the operational lifetime of underwater communication systems. Several studies have proposed algorithms for optimizing node placement and connectivity in underwater environments. Felamban et al. [11] investigated node placement strategies for underwater Wireless Sensor Networks (WSNs) to address the unique challenges of underwater acoustic communication, such as high transmission loss and limited coverage. They formulated the problem as a nonlinear mathematical optimization model to minimize transmission loss while ensuring effective 3D coverage using a truncated octahedron topology. Their approach was validated through both mathematical modeling and simulations, showing less than 10% error between the two. Similarly, Alam et al. [12] explored the topology control problem in 3D underwater sensor networks, addressing the unique challenges posed by node deployment in underwater environments. For sparse networks, they proposed an approach to minimize the number of robust, location-specific nodes while ensuring full coverage and connectivity. For dense networks, they introduced a dynamic topology control algorithm that activates a subset of randomly deployed, failure-prone nodes to conserve energy while maintaining coverage and connectivity. Although both studies focus on initial infrastructure design for non-SDN underwater networks, they provide valuable insights into topology design that can inform future research in SDN-based IoUT environments.

Choudhary et al. [14] analyzed different node deployment strategies, exploring their complexity and providing a comprehensive literature survey on deployment issues and performance evaluations of various deployment algorithms. They examined the specific challenges of deploying sensor nodes in UWSNs, highlighting the differences between 2-D and 3-D deployment environments. Their study provides a thorough survey of existing node deployment strategies, focusing on the complexities introduced by water currents, mobility, and communication delays. Benisto and Sukumaran [15] explored node deployment in UWSNs, emphasizing strategies to improve communication range, signal amplification, and data reliability. They noted that the unique 3-D properties of UWSNs render many terrestrial deployment strategies inapplicable due to challenges like node mobility from water currents and communication delays. Their analysis of deployment methodologies and extensive literature review provides valuable insights for optimizing node placement, enhancing communication efficiency, and addressing the challenges of underwater environments. Lohan et al. [16] proposed a novel 3D topology design for underwater sensor networks, aiming to achieve full coverage with the minimum number of sensor nodes while considering boundary effects. The authors analytically derive the optimal distance between nodes based on sensing and communication ranges and provide placement coordinates for effective deployment. However, while the proposed method is described and analyzed using MATLAB, it lacks implementation in a simulator to thoroughly investigate energy efficiency and quality of service (QoS), limiting its practical applicability and real-world validation.

While the aforementioned works have laid a foundation for topology design in underwater networks, their applicability to SDN-based IoUT systems remains limited. SDN’s centralized control capabilities enable dynamic resource allocation, global network awareness, and adaptive routing, making it an ideal paradigm for underwater networks. However, the integration of SDN with topology design has not been thoroughly explored, leaving a critical gap in understanding the interplay between topology structure and Quality of Service (QoS) in SDN-enabled IoUT environments. To the best of our knowledge, this is the first work to comprehensively investigate topology design in SDN-based IoUT environments. By leveraging SDN’s centralized control and dynamic routing capabilities, we evaluate the impact of 3D geometric topologies on QoS metrics. This study provides novel insights into the trade-offs between topology structures and their implications for efficient underwater network design, addressing a critical gap in the current body of research.

Despite the valuable foundations established by previous studies in topology design for underwater networks, their applicability to SDN-based IoUT systems remains limited. In particular, most existing works focus mainly on coverage or connectivity aspects and rarely investigate how 3D geometric deployment structures interact with centralized SDN control, shortest-path routing, and multiple Quality of Service (QoS) objectives within a unified evaluation framework. Given the global network visibility and flexible routing capabilities enabled by SDN, understanding the relationship between topology structure and network performance becomes increasingly important for efficient underwater communication systems. Therefore, this work addresses this gap by presenting a systematic SDN-based comparative analysis of several 3D geometric topologies under consistent routing and simulation settings. The study highlights the trade-offs among coverage efficiency, QoS performance, and deployment cost in SDN-enabled IoUT environments.

Table 1 provides a concise comparative overview of representative studies on underwater wireless sensor networks and topology control approaches. The table highlights the network type, primary objectives, employed methodologies, evaluated QoS metrics, topology characteristics, and key limitations of each study. While the reviewed works contribute valuable insights into coverage optimization, transmission reliability, and deployment strategies in underwater environments, most of them rely on static network topologies and focus on a limited set of performance metrics. In particular, non-SDN-based approaches often lack adaptability to dynamic network conditions and do not comprehensively address end-to-end QoS requirements. In contrast, the proposed SDN-based IoUT architecture adopts a dynamic topology design and integrates multiple QoS metrics, including delay, packet delivery ratio, and network lifetime, thereby addressing several limitations observed in existing solutions.

**Table 1:** Comparison of related work.

Study	Network Type	Primary Objective	Limitation
Felambo et al. [11]	Underwater WSN – Static	Minimize transmission loss	Focus on transmission loss
Alam et al. [12]	D3 UWSN – Static	sparse and dense networks	Focus on energy
Choudhary et al. [14]	Non-SDN UWSN – Static	Survey of deployment strategies	No real-world validation
Benisto and Sukumar [15]	Non-SDN UWSN – Static	Communication range and reliability	No QoS evaluation
Lohan et al. [16]	Non-SDN UWSN – Static	Full coverage with minimum nodes	No energy or QoS analysis
Proposed architecture	SDN-based IoUT – Dynamic	Topology design in SDN-enabled IoUT	Implementation complexity and SDN infrastructure dependency

## Materials and methods

In this paper, to evaluate the impact of various topology design algorithms, we utilize an SDN-based architecture. The adoption of SDN in IoUT networks is a well-established approach and has been extensively studied and validated by researchers in different contexts [7,17,18]. In the proposed method, it is initially assumed that the coordinates of the nodes in the 3D underwater environment have been determined based on one of the four topology design methods and have been deployed at their respective locations. Subsequently, the network controller takes responsibility for route calculation and network management.

before the path computation operation, a network topology graph must first be created. For this purpose, the underwater nodes are configured to periodically transmit their three-dimensional coordinate information and remaining battery energy to the controller. The period of transmission of this information is determined by the network administrator. The information is transmitted in broadcast fashion so that it ultimately reaches the controller. Therefore, it first calculates the delay of each link using the following Eq. 1 as follows:


$$\begin{array}{c} d_{ab}=\sqrt{(x_{a}-x_{b}{)}^{2}+(y_{a}-y_{b}{)}^{2}+(z_{a}-z_{b}{)}^{2}} \end{array}$$ (1)

where $d_{ab}$ is the Euclidean distance between the nodes in 3D environment. To compute the reliability of transmission of a data packet with the length of *n* between two neighbour nodes, the controller uses the following underwater communication channel equations:


$$\begin{array}{c} P_{successful}^{n,(a,b)}(\gamma )=[1-BER(\gamma ){]}^{n}=\\ {}[P_{successful}^{1}(\gamma ){]}^{n}=\{\frac{1}{2}+\frac{1}{2}\sqrt{\frac{10^{\gamma /10}}{1+10^{\gamma /10}}}{\}}^{n} \end{array}$$ (2)

where γ is the Signal-to-Noise Ratio (SNR) and *BER(*γ) is the Bit Error Rate of the underwater channel, respectively.

### Node coordinate calculation phase

Topology design in underwater networks involves the strategic placement of nodes to achieve optimal connectivity, coverage, and energy efficiency. Below, we provide a detailed explanation of the three topology design algorithms considered in this study.

#### Cubic.

The CB topology divides the underwater environment into a grid of cubic cells, where each node is positioned at the center of a cube. As depicted in Fig 1, a Cube is the basic 3D structure composed of six square faces, offering simple and uniform connectivity among nodes placed at its vertices. This design provides a regular and structured distribution of nodes in 3D space, ensuring consistent coverage and reducing the complexity of the network. The cubic arrangement allows for straightforward routing and efficient spatial organization, as each node is equidistant from its neighbors. Mathematically, the coordinates of the nodes in the cubic topology can be calculated as follows [12].


$$\begin{array}{c} R=min(r_{c}\frac{\sqrt{3}}{2},r_{s}) \end{array}$$ (3)

$$\begin{array}{c} \left(x+u\frac{2R}{\sqrt{3}},y+v\frac{2R}{\sqrt{3}},z+w\frac{2R}{\sqrt{3}}\right) \end{array}$$ (4)

**Fig 1:**
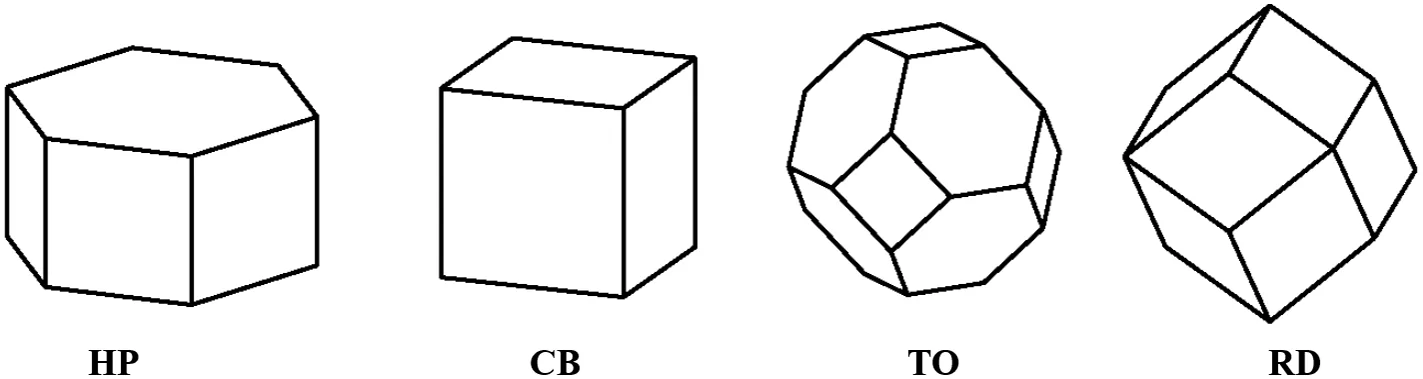
3D structure of the cells for different geometric shapes.

where $r_{c}$ is the communication range and $r_{s}$ is the sensing range of each node, u,v,w∈Z (set of integers), and (*x*,*y*,*z*) is an arbitrary reference point, respectively. In this paper, it is important to distinguish between the sensing range and the communication range of underwater sensor nodes. The sensing range refers to the local area around the node within which it can observe environmental data (e.g., pressure, temperature). In contrast, the communication range defines how far a node can transmit or receive signals from other nodes in the network. Typically, the communication range in underwater acoustic networks is significantly larger than the sensing range due to the nature of acoustic propagation. Understanding this distinction is essential for designing efficient coverage and connectivity strategies.

#### Hexagonal prism.

The HP topology divides the underwater environment into hexagonal columns, providing a uniform distribution of nodes in 3D space. Each prism is formed by stacking hexagonal layers vertically. As shown in Fig 1, a Hexagonal Prism is a 3D topology consisting of two hexagonal bases connected by rectangular sides, providing balanced horizontal and vertical connectivity. This structure ensures efficient coverage and connectivity while minimizing overlap. In the Hexagonal Prism topology, nodes are placed at the centers of the hexagonal prisms to ensure uniform coverage and connectivity in the 3D underwater environment. Mathematically, the coordinates of nodes in the hexagonal prism topology can be defined as [12]:


$$\begin{array}{c} a=min(\frac{r_{c}}{\sqrt{3}},r_{s}\frac{\sqrt{2}}{\sqrt{3}}) \end{array}$$ (5)

$$\begin{array}{c} b=min(2\sqrt{r_{s}^{2}-a^{2}},r_{c}) \end{array}$$ (6)

$$\begin{array}{c} \left(x+\frac{3ua}{\sqrt{2}},y+ua\frac{\sqrt{3}}{2}+va\sqrt{3},z+wb\right) \end{array}$$ (7)

Where the variables are defined, as well as in Eq. 4.

#### Rhombic dodecahedron.

The RD topology partitions the underwater environment into cells shaped like rhombic dodecahedra, which are space-filling polyhedra. Nodes are placed at the vertices of these dodecahedra to optimize coverage. As illustrated in Fig. 1, a polyhedron with 12 rhombic faces, enabling high spatial efficiency and uniform neighbor distribution in 3D networks. The coordination of each node using RD algorithm is given by [12]:


$$\begin{array}{c} R=min(\frac{r_{c}}{\sqrt{3}},r_{s}) \end{array}$$ (8)

$$\begin{array}{c} \left(x+\frac{2(2u+w)R}{\sqrt{5}},y+\frac{2(2v+w)R}{\sqrt{5}},z+\frac{2wR}{\sqrt{5}}\right) \end{array}$$ (9)

Where the variables are defined, as well as in Eq. 4.

#### Truncated octahedron.

The TO topology divides the underwater space into cells resembling truncated octahedrons, another space-filling structure. This topology provides high connectivity and fault tolerance. Nodes are placed at the vertices of the truncated octahedrons, ensuring robust communication. As depicted in Fig 1, the truncated octahedron is a space-filling polyhedron with 8 regular hexagonal faces and 6 square faces, offering efficient 3D tessellation and improved connectivity with minimal overlap. Mathematically, the coordinates of the nodes in the truncated octahedral topology can be calculated as follows [12].


$$\begin{array}{c} R=min(\frac{r_{c}\sqrt{5}}{4},r_{s}) \end{array}$$ (10)

$$\begin{array}{c} \left(x+\frac{(2u+w)R}{\sqrt{2}},y+\frac{(2v+w)R}{\sqrt{2}},z+wR\right) \end{array}$$ (11)

Where the variables are defined similarly to those in Eq. 4. Fig 1 illustrates the 3D structure of the cells for the mentioned geometric shapes.

#### Route calculation and management phase.

In this paper, we assume that the SDN controller is positioned at the water surface, centrally located within the network topology. Beyond its primary role as an SDN controller, it also functions as a data aggregator (i.e., Sink), collecting information from underwater nodes and transmitting it to the coastal station. The controller periodically broadcasts messages to all network nodes at predefined intervals, requesting them to report their current energy levels and three-dimensional coordinates. Upon receiving these broadcast messages, all active nodes (those whose battery levels have not yet reached zero) transmit their energy status and current coordinates to the controller. It is worth mentioning that, once an active node is not within one hop of the SDN controller, it transmits its state information (energy levels and coordinates) via multi-hop communication. Intermediate nodes forward the data in a broadcast manner, ensuring reliable delivery of status updates to the controller. Using the collected data, the controller constructs a network graph and employs Dijkstra’s algorithm to compute hop-count-based shortest paths between the traffic source nodes and the sink node. In this study, hop count is used as the routing metric for shortest-path computation. The calculated paths are then disseminated to the respective nodes along these routes, instructing them to forward packets toward the sink according to the specified paths. This process is repeated periodically until the network reaches the end of its operational lifetime. In our proposed architecture, traffic-generating nodes located at the seabed begin transmitting packets toward the sink following the calculated paths. By leveraging this approach, the study evaluates how different topology design algorithms influence network performance, including packet delivery, energy efficiency, and delay metrics, under a unified SDN architecture. Algorithm 1 illustrates our proposed architecture. In this algorithm, the broadcast time slot T determines how frequently the SDN controller gathers status information (e.g., energy level and position) from the active nodes. This parameter is configurable and can be adjusted by the network administrator based on application requirements. A smaller T leads to more up-to-date and accurate global network information at the controller, enabling better routing decisions. However, it also results in higher energy consumption due to the increased frequency of control message exchanges. On the other hand, a larger T reduces communication overhead and conserves node energy but may cause the controller to base its decisions on outdated information, potentially degrading routing efficiency.

**Algorithm 1 Proposed Method for SDN-based IoUT with topology design Algorithms**

1:  **Input:** Precomputed 3D coordinates of nodes based on topology design algorithm, predefined broadcast interval *T*

2:  Place nodes in the underwater environment using precomputed 3D coordinates.

3:  Position SDN controller at the water surface.

4:  **while** Network is operational **do**

5:   Controller broadcasts a message to all nodes requesting energy levels and coordinates.

6:   **for all** Nodes i∈N **do**

7:    **if** Node *i* is active (battery >0) **then**

8:     Node *i* sends its energy level $E_{i}$ and coordinates $\left(x_{i},y_{i},z_{i}\right)$ to the controller.

9:    **end if**

10:   **end for**

11:   Controller constructs the network graph *G* = (*V*, *E*) using received data

12:   **for all** Traffic source nodes src∈S **do**

13:    Compute shortest paths $P_{src\to sink}$ using Dijkstra’s algorithm.

14:    Disseminate calculated paths $P_{src\to sink}$ to respective nodes.

15:    Nodes forward packets to the sink along paths $P_{src\to sink}$.

16:   **end for**

17:  **end while**

As illustrated in Fig 2 the SDN controller periodically collects residual energy and location information from active underwater nodes, constructs the network graph, computes optimal routing paths for each traffic source using Dijkstra’s algorithm, and disseminates these paths to the nodes for data forwarding to the sink.

**Fig 2:**
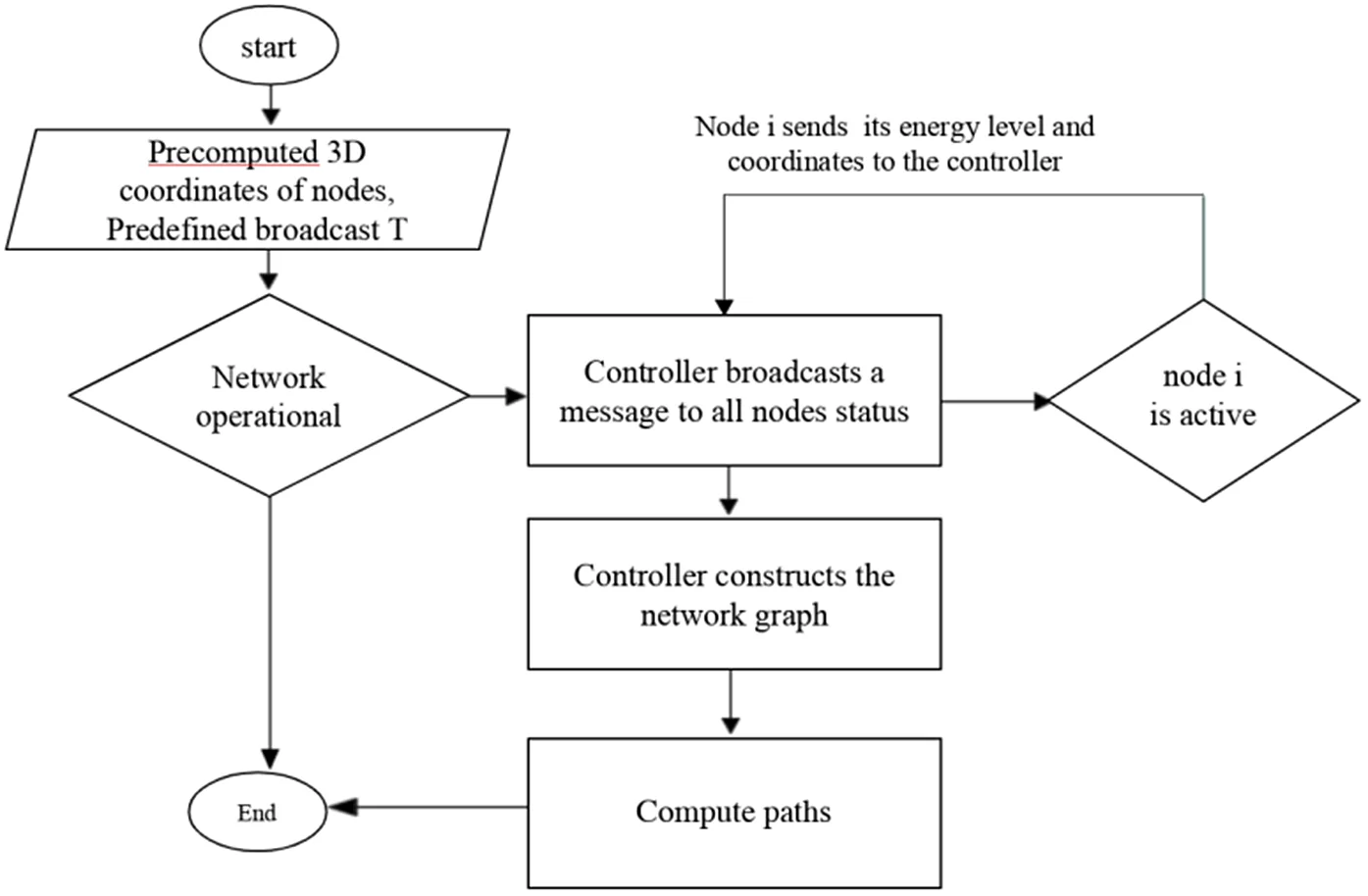
Proposed Method for SDN-based IoUT with topology design Flowchart.

## Performance evaluation

To assess the efficacy of the proposed topology design algorithms for SDN-based IoUT networks, we conduct a series of simulations comparing the performance of different topologies against the benchmark Grid deployment. In the Grid deployment, nodes are arranged in a grid formation with distances between them approximately equal to their sensing range, ensuring a uniform spatial distribution.

A novel simulation framework was developed for this study, tailored to model the hybrid nature of underwater and terrestrial communications. The simulator integrates SDN architecture, leveraging Python libraries such as NetworkX [19] for network modeling and Threading [20] for handling concurrent processes. The simulation differentiates between underwater communication, which uses acoustic signals, and terrestrial communication, which employs wireless radio signals, to accurately represent the IoUT environment.

To ensure the reliability and fairness of our analysis, we compare the proposed method against Dijkstra routing protocol that uses hop-count as a metric, a standard practice in many network routing schemes [21]. Because hop-count shortest-path routing is a fundamental and widely adopted mechanism in network performance evaluation literature, it is employed in this study as a baseline routing strategy. This choice provides a standardized benchmark for rigorously assessing the efficiency of the proposed geometric topology designs.This established baseline ensures that our comparative analysis of QoS metrics is grounded in a well-validated routing paradigm common to existing research in this domain. By focusing on this comparison, we can evaluate the impact of advanced SDN-driven routing methods on critical QoS parameters in IoUT environments.

The simulation tests were conducted on a high-performance machine featuring an Intel Core i7-5930k CPU and 16 GB of memory. We modeled a 3D underwater environment of size 2000m×2000m×2000m. Of all the nodes, 20 nodes are designated as traffic-generating nodes, and the SDN controller, located at the center of the topology, also functioned as a sink node, collecting data from the underwater devices every 10 seconds and calculates paths, and informs them to the relevant nodes.

Traffic was modeled using a Poisson distribution, which is frequently used in network simulations due to its capability to represent random packet arrivals [22]. The simulation parameters are designed to reflect realistic underwater conditions, as detailed in Table 2.

**Table 2:** Simulation Parameters.

Parameters	Value
Initial Energy	10000 joule
Sink to onshore station distance	2 Km
Sink to onshore station Communication	IEEE 802.11/b (DBPSK)
Number of Underwater Sensors	Different for each topology design algorithm
Number of Senders	20
Communication Range ($r_{c}$)	300 meter
Sensing Range ($r_{s}$)	$\frac{\sqrt{5}r_{c}}{4}$ meter
Simulation Area	2000*2000*2000 (3D)
Simulation Time	300 Seconds
Broadcast Packet size	60 byte
Acoustic signal speed	1500 meters/sec
Frequency	25 KHz
Tx power consumption	0.02 mWatt
Rx power consumption	0.01 mWatt
Bandwidth	10 kbps
Data Packet Size	200 bytes
Packet Rate	1 to 5 packets/sec

### Metrics and evaluation metrics

The performance of the network is evaluated based on the following metrics:

- **Total Energy Consumption:** This metric measures the overall energy used by the network during the simulation period. It is critical for IoUT systems as energy efficiency directly impacts the network’s sustainability and operational lifetime.
- **Network Lifetime:** This metric represents the duration until the sink node no longer receives data from any source nodes in the network. A shorter network lifetime suggests that intermediate nodes have exhausted their energy, leading to a breakdown in data transmission paths between the source nodes and the sink. This is a critical indicator of the network’s sustainability and energy efficiency.
- **Average End-to-End Delay:** This metric captures the time it takes for a packet to travel from the source to the destination, considering all hops. Lower delay is vital for ensuring real-time communication in IoUT networks.
- **Total Received Data:** This metric refers to the total amount of data received by the sink node from all source nodes in the network. A higher volume of data received during the simulation indicates better network performance, as it reflects the network’s ability to effectively transmit data under the given conditions.
- **Number of Required Nodes:** The number of nodes required to cover the entire network is a critical parameter that can significantly impact the financial cost of network deployment. The fewer the nodes needed to ensure full coverage, the more cost-effective the network will be economically.
- **Packet Delivery Ratio (PDR):** This metric is a key performance metric used to evaluate the efficiency and reliability of communication in sensor networks. It is defined as the ratio of the number of data packets successfully received by the sink node to the total number of packets sent by the source nodes. A higher PDR indicates better network performance, demonstrating that the routing mechanism effectively delivers data with minimal loss.

Simulation results for the number of nodes required for deployment in a 3D underwater environment with dimensions of 2000 × 2000 × 2000 as illustrated in Fig 3 reveal that the GRID method requires the highest number of nodes. This is because the nodes are evenly distributed in the 3D space based on their sensing range, making this method costly in terms of financial and deployment expenses. However, it is primarily used as a baseline for comparing other methods. Among the evaluated methods, RD achieves the best results, requiring only 658 nodes. This is followed by the HP method with 861 nodes, while the TO and CB methods require significantly more nodes. This result highlights an important geometric trade-off in topology design. The superior performance of the RD topology in terms of node count can be attributed to its space-filling structure, which provides efficient volumetric coverage with less redundancy than the GRID and CB arrangements. In other words, RD achieves a more favorable balance between coverage completeness and deployment density. This reduction in the number of required nodes is not only economically beneficial, but also affects subsequent network behavior, since fewer deployed nodes generally reduce control overhead, maintenance complexity, and the probability of unnecessary relaying.

**Fig 3:**
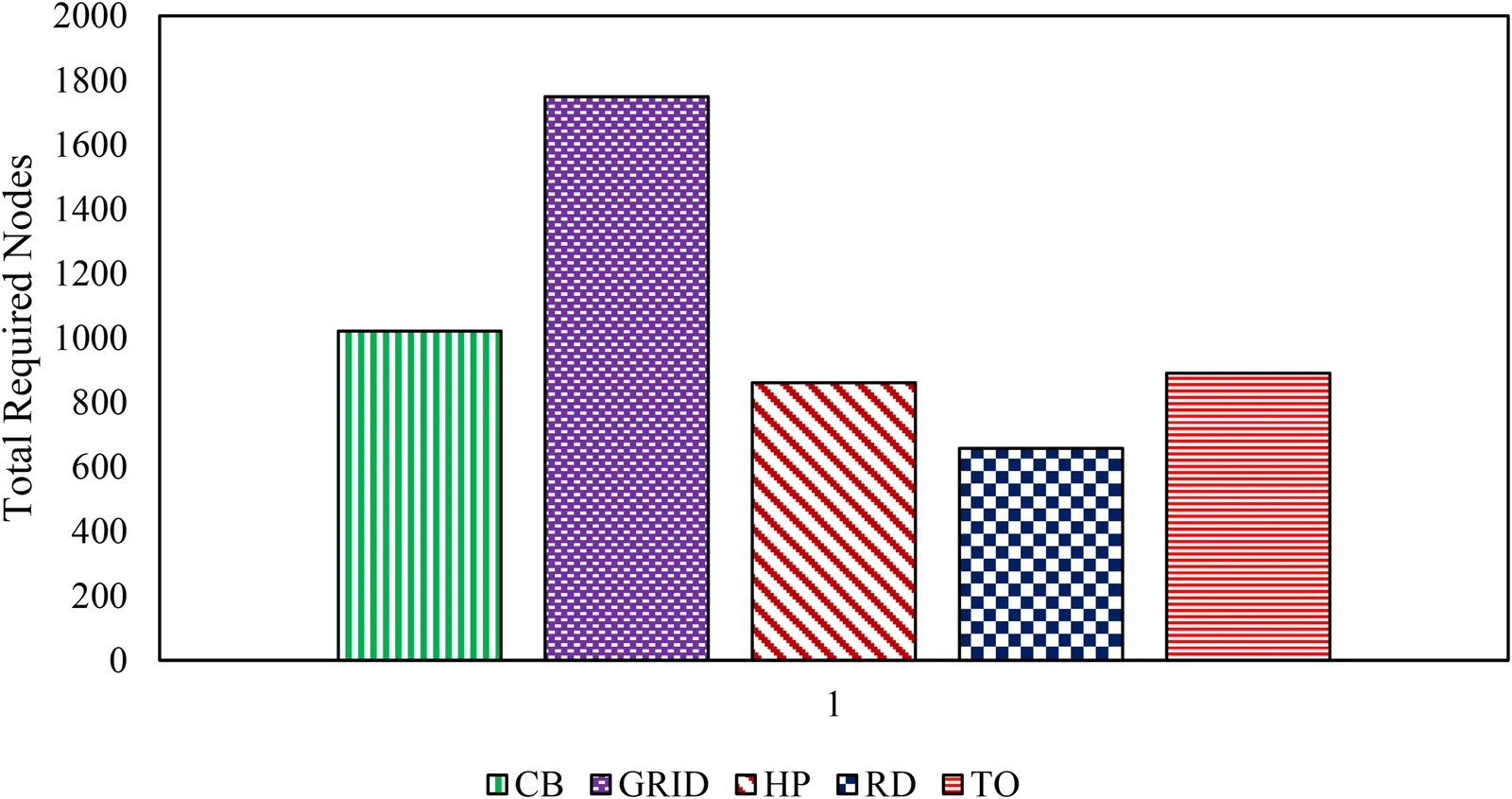
Total Number of Required Nodes.

Fig 4 illustrates the simulation results for the total received data metric under varying packet sizes and transmission rates. As shown, the GRID and RD deployment methods enable the sink node to collect the maximum amount of data. This is because, in the GRID method, nodes are distributed with redundancy across the environment, ensuring the availability of alternative routes even if some nodes fail. Similarly, in the RD method, nodes are deployed in a manner that maintains consistent connectivity with their neighbors, enabling more data to reach the sink node. Conversely, the CB and TO deployment methods show the poorest performance in terms of data delivery to the sink, rendering them less suitable for this metric.

**Fig 4:**
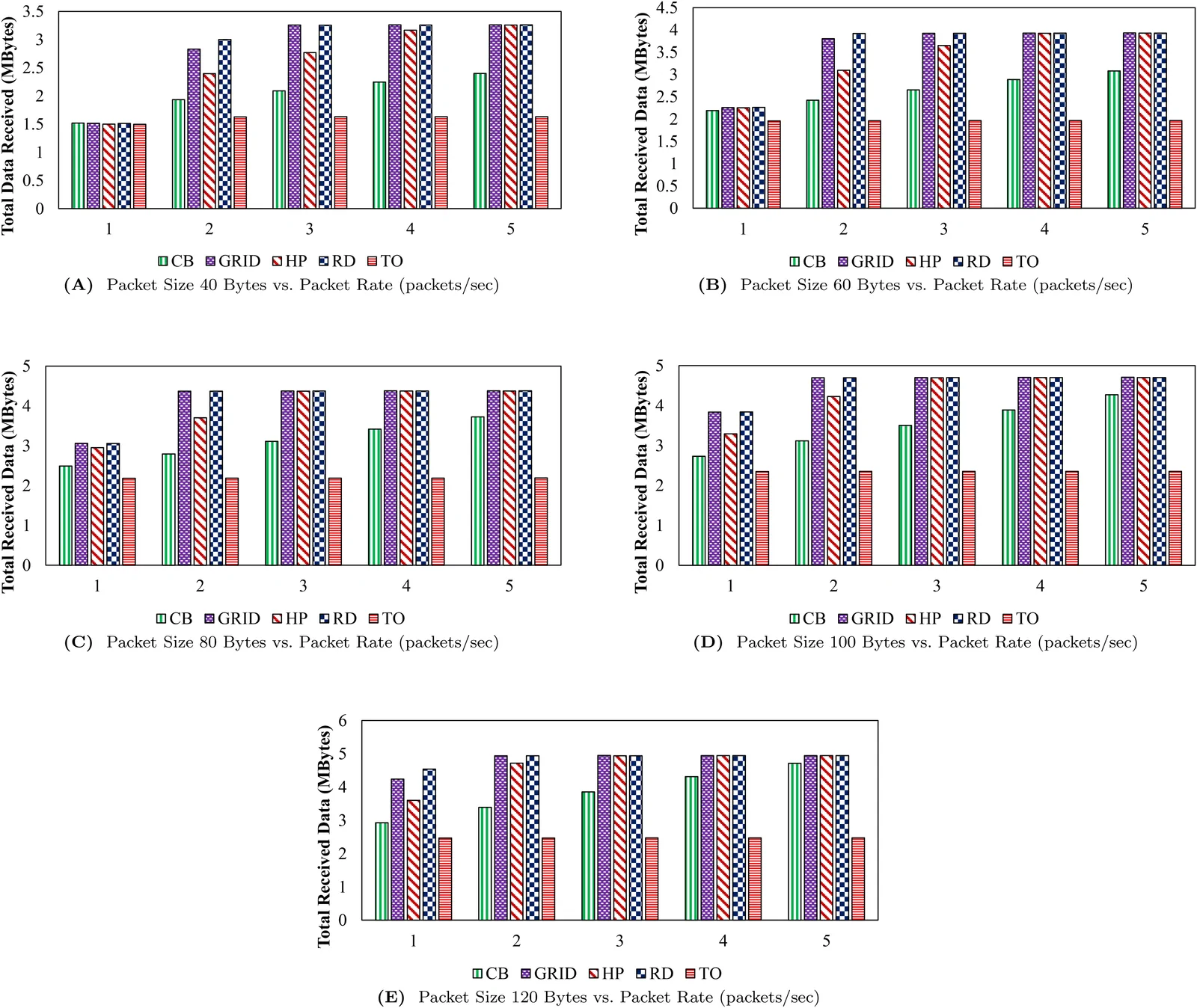
Total Received Bytes vs. Packet Rate.

Fig 5 highlights the total energy consumption across different packet sizes and transmission rates. The GRID method consumes the most energy due to the presence of redundant nodes, followed by the HP method. On the other hand, the RD and TO methods exhibit the best energy efficiency, consuming the least energy among all methods. The energy results further emphasize the cost of structural redundancy. In the GRID topology, the larger number of deployed nodes increases not only the amount of data forwarding activity, but also the control overhead associated with periodic state reporting and path dissemination in the SDN framework. By contrast, RD benefits from a reduced node population while still maintaining sufficient connectivity, which leads to lower cumulative transmission and reception costs. Therefore, the energy advantage of RD is not merely a consequence of fewer nodes, but also of more efficient topology-driven utilization of network resources.

**Fig 5:**
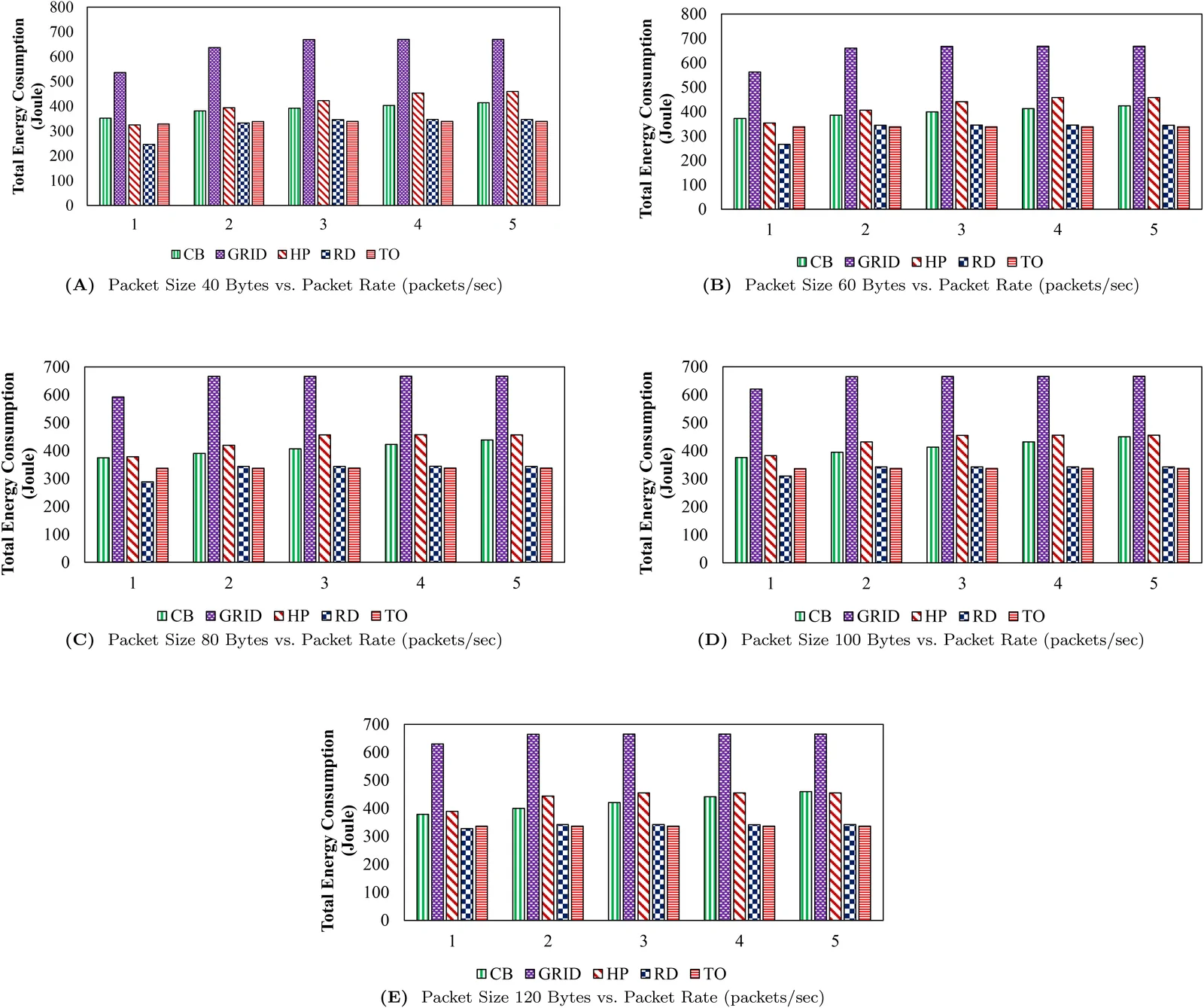
Total Energy Consumption vs. Packet Rate.

Fig 6 presents simulation results for the network lifetime metric under varying packet sizes and transmission rates. These results demonstrate that the GRID and RD methods achieve the highest network lifetime. In scenarios with low transmission rates, the network lifetime even reaches the simulation duration of 300 seconds. As discussed earlier, the well-distributed nodes in the GRID and RD methods ensure consistent routing paths from source nodes to the sink, allowing for prolonged data transmission. However, in other methods, some nodes may deplete their energy prematurely, causing network partitioning and preventing data from reaching the sink. While the HP method shows moderate performance in terms of network lifetime, the CB and TO methods exhibit the weakest results, making them unsuitable for underwater environments. From an analytical perspective, network lifetime is strongly influenced by how evenly the forwarding burden is distributed across the topology. In both GRID and RD deployments, connectivity remains sufficiently stable to prevent early isolation of traffic-generating nodes. However, the RD topology is particularly attractive because it achieves a lifetime comparable to GRID while requiring substantially fewer nodes. This indicates that lifetime improvement is not solely dependent on deployment density, but also on the geometric efficiency of the topology in preserving sustainable multi-hop paths. In contrast, when the topology leads to bottleneck nodes or reduced path diversity, energy depletion occurs more rapidly and network fragmentation appears earlier.

**Fig 6:**
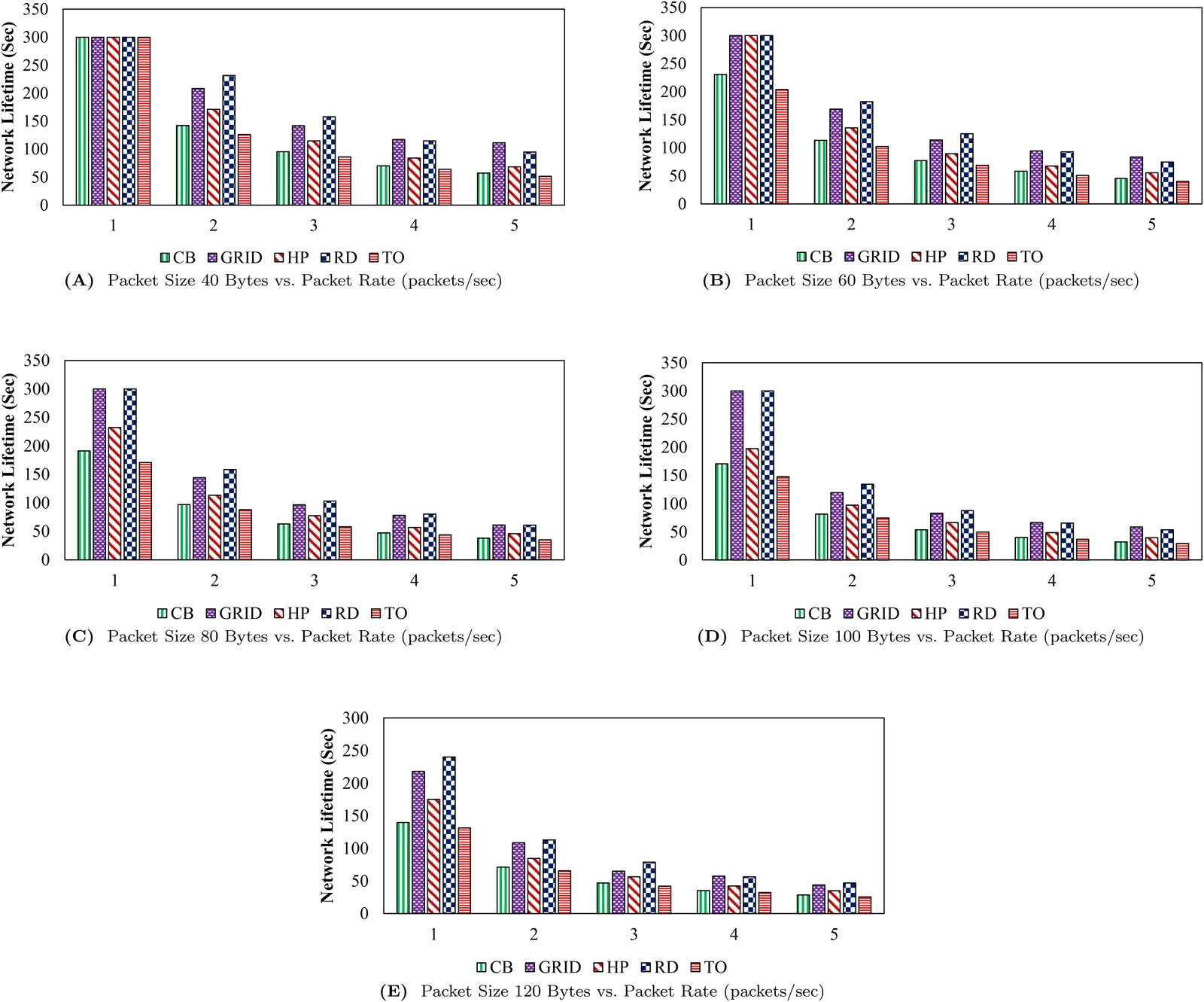
Network Lifetime vs. Packet Rate.

As depicted in Fig 7, simulation results for the end-to-end delay metric reveal that the GRID method exhibits the highest delay among all methods. This is attributed to the presence of redundant nodes, which increases the number of hops between the source and sink nodes. In contrast, the RD method, with its optimized node deployment, reduces the number of hops required to establish paths between source and sink nodes. The TO method shows average performance for delay, while the HP and CB methods demonstrate the worst delay performance among the evaluated methods. These results demonstrate that delay is closely related to the interplay between path length and topology efficiency. Although denser deployments may improve connectivity, they do not necessarily minimize delay, especially when packet forwarding traverses a larger number of intermediate nodes. The RD topology appears to offer a more favorable compromise, preserving connectivity while limiting excessive hop expansion. This observation is particularly relevant for SDN-based IoUT systems, where routing decisions are centrally computed and the underlying topology directly influences the quality of the selected paths.

**Fig 7:**
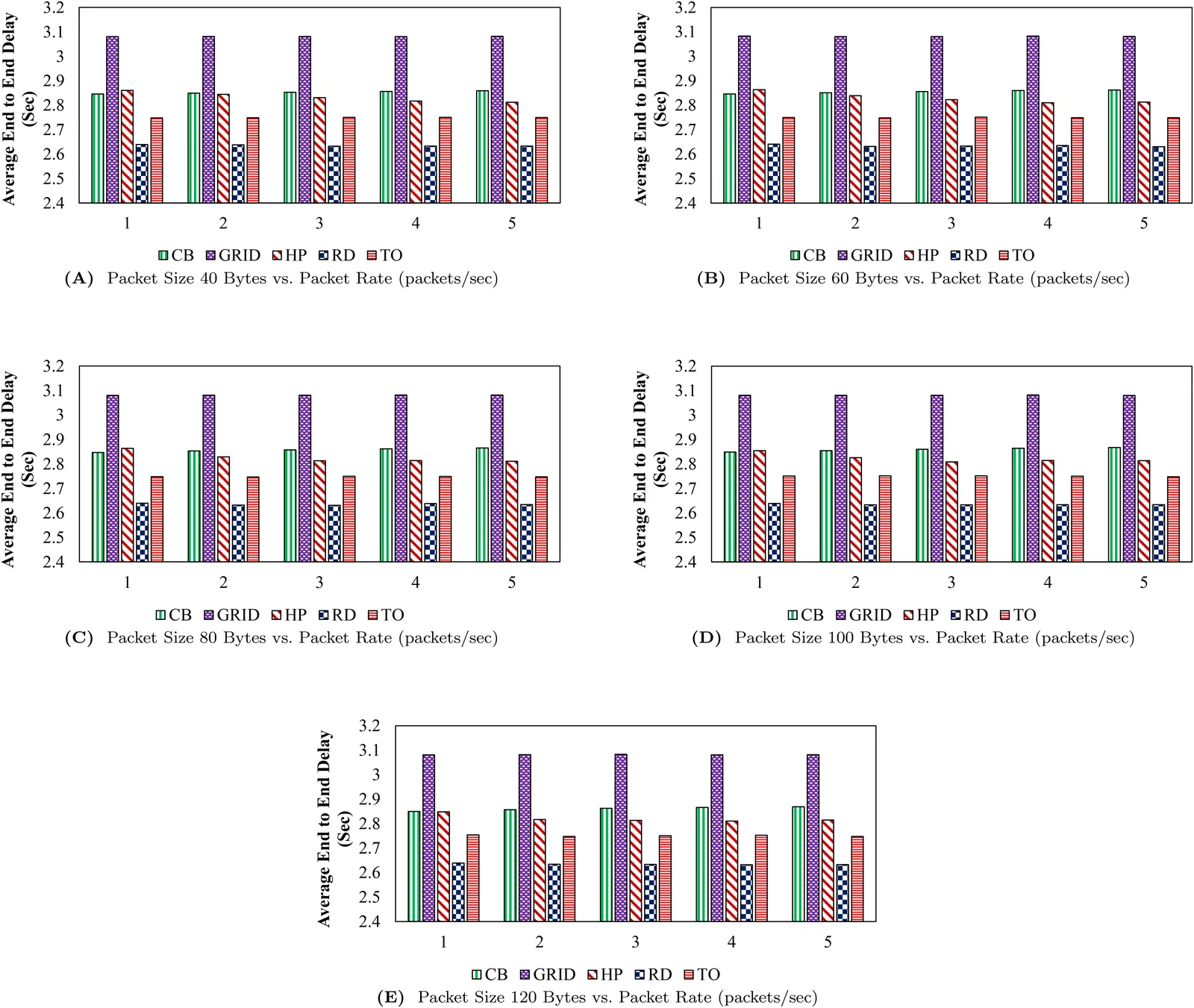
Average End-to-End Delay vs. Packet Rate.

Fig 8 shows PDR under different transmission rates and packet sizes to assess the reliability and effectiveness of data delivery in various deployment strategies. The results show a strong correlation between network lifetime, as depicted in Fig 6, and PDR values. Specifically, the GRID and RD methods, which demonstrated the longest network lifetimes, also achieved the highest PDR values. This outcome is expected, as a longer network lifetime allows more sensor nodes to remain active and participate in consistent data forwarding, thereby ensuring a higher number of successfully delivered packets. In contrast, the CB and TO methods, which exhibited the shortest lifetimes, also suffered from low PDR values. As nodes deplete their energy and disconnect from the network, packet losses increase and connectivity to the sink node is disrupted. The HP method shows intermediate results for both metrics, highlighting its partial effectiveness in maintaining a reliable transmission environment. Moreover, the results presented in Fig 8 clearly indicate that as the packet transmission rate increases, the PDR significantly decreases. This decline is primarily due to the faster depletion of energy in the sensor nodes, which leads to network fragmentation and the inability to establish routing paths between the traffic source nodes and the sink. The PDR results also reinforce the broader relationship between topology stability and communication reliability. Topologies that preserve connectivity for longer durations naturally maintain a higher probability of successful packet delivery. However, this should not be interpreted as a purely connectivity-driven outcome. The structural arrangement of nodes also affects the robustness of forwarding paths under increasing traffic rates. In this regard, RD demonstrates that an efficient geometric deployment can simultaneously support reliability, reduce energy expenditure, and limit the number of required nodes, making it more suitable for practical SDN-enabled IoUT scenarios than approaches that rely heavily on redundancy.

**Fig 8:**
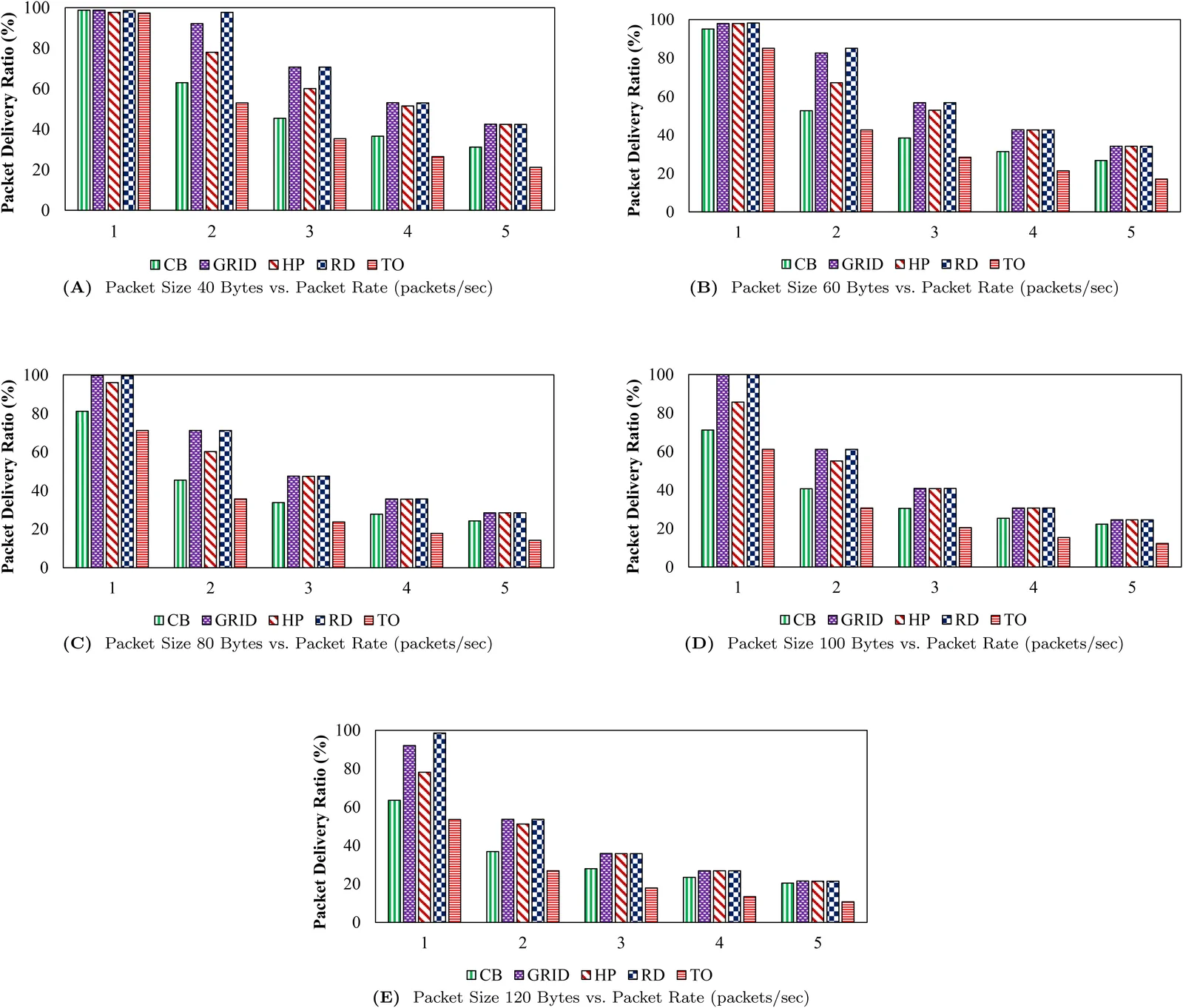
Packet Delivery Ratio vs. Packet Rate.

Based on the above metrics, the RD deployment method is identified as the most suitable and efficient approach across all evaluation criteria. Its lower node deployment requirements also contribute to reduced overall network implementation costs. The HP method ranks second, offering moderate deployment costs and favorable performance in key metrics such as network lifetime and total received data. However, the CB and TO methods show weak to below-average results across all metrics and require a higher number of nodes, making them less preferable for underwater environments. Overall, the simulation results indicate that topology performance in SDN-based IoUT networks is governed by a fundamental trade-off among deployment density, path diversity, routing efficiency, and energy sustainability. Dense topologies such as GRID improve robustness through redundancy, but they also increase delay, energy consumption, and deployment cost. In contrast, the RD topology achieves a more balanced performance profile by reducing the number of required nodes while preserving stable connectivity and efficient routing behavior. This suggests that, in underwater SDN environments, geometric efficiency may be more beneficial than simple deployment density when the objective is to jointly optimize QoS and implementation cost.

## Discussion

Although this paper presented the node placement strategies based on geometric arrangements, it is important to acknowledge that these methods alone may not suffice in real-world scenarios. Deploying nodes in harsh underwater environments, such as seas and oceans, faces numerous challenges, including unpredictable terrain, equipment constraints, and environmental disturbances. Moreover, after deployment, underwater nodes are subject to constant motion due to turbulence and water currents, which may significantly impact network performance. To mitigate this, effective anchoring mechanisms are required to minimize node displacement and maintain stable network connectivity. The methodologies evaluated in this paper, like many others in the literature, are based on idealized assumptions and optimized conditions, which may not always be achievable in real-world applications. Nonetheless, these methods and their simulation results provide valuable upper bounds for key QoS metrics, such as throughput, energy consumption, and delay. These upper bounds can serve as benchmarks for assessing the feasibility of implementing underwater networks in specific scenarios. By understanding the limitations and trade-offs, researchers and engineers can make informed decisions about whether to proceed with the deployment of underwater IoUT systems under given constraints. To enhance scalability for larger networks, the capacity of the controller should be increased. However, scalability was not the primary focus of our current work; our main goal was instead to determine the minimum required number of nodes within the three-dimensional environment.

Finally, it should be noted that centralization in SDN is a logical rather than a physical concept. Network capacity can be scaled by deploying multiple coordinated controllers instead of a single one. This approach improves scalability and reduces control overhead in large IoUT networks. The proposed SDN-based framework may face scalability challenges in large-scale networks, as increasing the number of deployed nodes results in higher control overhead and computational load at the controller. Consequently, large-scale IoUT deployments may require hierarchical or multi-controller architectures to achieve improved scalability.

## Conclusion

In this paper, we analyzed the performance of four 3D topology design algorithms—Cubic (CB), Hexagonal Prism (HP), Rhombic Dodecahedron (RD), and Truncated Octahedron (TO)—for optimizing QoS in SDN-based IoUT systems. Among these, the RD topology consistently outperformed others, achieving high data delivery and network lifetime while requiring the fewest nodes for deployment. Its optimized spatial arrangement ensured stable connectivity and efficient routing, resulting in lower energy consumption and reduced implementation costs. In comparison, the HP topology showed moderate performance, providing acceptable network lifetime and energy efficiency but requiring more nodes than RD. On the other hand, CB and TO demonstrated weak results across most metrics, with higher energy consumption, shorter network lifetime, and suboptimal node deployment efficiency. These findings highlight RD as the most effective topology for SDN-enabled IoUT systems, balancing performance and cost. Future research could focus on incorporating dynamic node mobility and adaptive routing to further enhance network reliability.
